# Supplementary material for: An individualized mosaic of maternal microbial strains is transmitted to the infant gut microbial community
Source: R Soc Open Sci. 2020 Apr 15;7(4):192200. doi: 10.1098/rsos.192200 (PMC7211887; doi:10.1098/rsos.192200)
Supplement: Figure S1. StrainPhlAn on the mothers-infants data set. [file rsos192200supp9.pptx]

## Slide 1
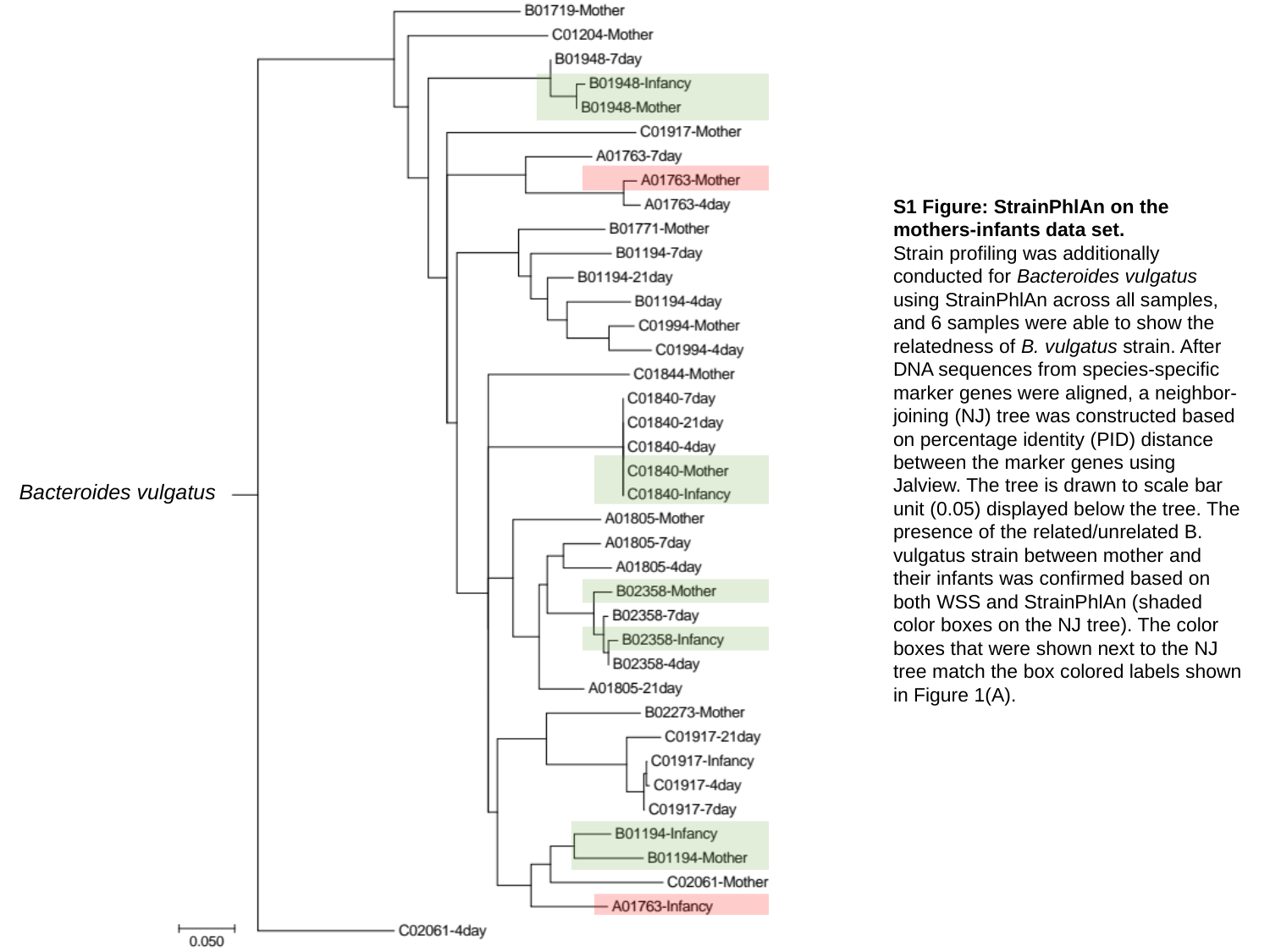

Bacteroides vulgatus
S1 Figure: StrainPhlAn on the mothers-infants data set.
Strain profiling was additionally conducted for Bacteroides vulgatus using StrainPhlAn across all samples, and 6 samples were able to show the relatedness of B. vulgatus strain. After DNA sequences from species-specific marker genes were aligned, a neighbor-joining (NJ) tree was constructed based on percentage identity (PID) distance between the marker genes using Jalview. The tree is drawn to scale bar unit (0.05) displayed below the tree. The presence of the related/unrelated B. vulgatus strain between mother and their infants was confirmed based on both WSS and StrainPhlAn (shaded color boxes on the NJ tree). The color boxes that were shown next to the NJ tree match the box colored labels shown in Figure 1(A).
